# The composition of MDSC-subpopulations PMN-like, M-like, and e-like MDSC is associated with the severity of infectious mononucleosis in pediatric patients

**DOI:** 10.3389/fimmu.2026.1729699

**Published:** 2026-03-30

**Authors:** Lorenz Mihatsch, Karin Bartl, Julia Lange de Luna, Pia Wallraven, Katrin Gerrer, Kirstin Mittelstraß, Josef Mautner, Elfriede Noessner, Ulrike Protzer, Uta Behrends, Tanja Bauer, Nina Körber

**Affiliations:** 1 MRI Chronic Fatigue Center for Young People (MCFC), TUM School of Medicine and Health, Children’s Hospital, Technical University of Munich, Munich, Germany; 2 Institute of Virology, TUM School of Medicine and Health, Technical University of Munich, Munich, Germany; 3 Helmholtz Munich Institute of Virology, Munich, Germany; 4 German Center for Infection Research (DZIF), Partner Site Munich, Munich, Germany

**Keywords:** Epstein-Barr virus, IM-complexity, IM-severity, infectious mononucleosis, myeloid-derived suppressor cells, pediatric cohort

## Abstract

**Background:**

Primary infection with the Epstein-Barr virus (EBV) in early childhood is often asymptomatic, whereas infection with EBV during adolescence or adulthood may lead to infectious mononucleosis (IM). Sometimes severe complications like hepatitis, spleen rupture, or myocarditis may appear during IM. Myeloid-derived suppressor cells (MDSC) are a heterogeneous group of regulatory immune cells with immunosuppressive activities. However, not much is known about the role of MDSC in IM. We assessed a clinical translational prospective study to examine the role of MDSC during the early and late phases of IM in pediatric patients.

**Method:**

We performed a longitudinal analysis of PMN-like, M-like, and e-like MDSC frequencies in 37 pediatric patients during the early (V1 < 28 days after symptom onset (T_onset_), V2 four to six weeks after T_onset_) and late phases (V3 = six to 12 months after T_onset_) of IM using flow cytometry-based analyses. In addition, we determined the correlation of frequencies of MDSC-like subpopulations with the complexity and severity of laboratory, clinical, and virological IM features.

**Results:**

We detected comparable frequencies of total MDSC-like cells during the course of IM (V1 median: 22.87%, V2 median: 16.10%, and V3 median: 16.58% of CD33+ cells, respectively). However, we observed temporal dynamics of the MDSC-like subpopulations PMN-like, M-like, and e-like MDSC during the early and late phases of IM. The frequency of PMN-like MDSC decreased significantly from V1 to V2 (*P < 0.001*) and from V1 to V3 (*P < 0.001*). In contrast, the frequency of M-like MDSC decreased significantly from V1 to V2 (*P* = 0.015) and increased from V2 to V3 (*P* < 0.001). e-like MDSC frequencies increased significantly from V1 to V3 (*P* < 0.001). We observed an increase in the proportion of PMN-like MDSC with the IM-complexity and severity laboratory score and ferritin levels.

**Conclusion:**

Our data highlights the rationale for in-depth analyses of MDSC subpopulations in further studies on IM and contributes to obtaining a more differentiated picture of the ongoing changes in MDSC-like cell distribution and the relationship between frequencies of MDSC-like cells and the complexity and severity of laboratory, clinical, and virological IM features.

## 1 Introduction

The Epstein-Barr virus (EBV) is a γ-herpesvirus that infects more than 90% of the human population and persists lifelong (1). Primary infection with EBV in early childhood is often asymptomatic (2), whereas infection with EBV during adolescence or adulthood may lead to infectious mononucleosis (IM) (2, 3). IM is usually a self-limiting disease that typically presents with symptoms like fever, tonsillopharyngitis, cervical lymphadenopathy, hepatosplenomegaly, and fatigue (1, 2, 4–6). Sometimes, severe complications like hepatitis, spleen rupture, or myocarditis, as well as neurological or hematological complications, may appear during IM (1, 2, 4–6). After IM, long-lasting symptoms such as fatigue may persist, and EBV is the main pre-pandemic trigger of post-infectious myalgic encephalomyelitis/chronic fatigue syndrome (ME/CFS) (7). Fulfillment of ME/CFS diagnostic criteria has been reported in 13% of adolescents six months post IM (8–11). ME/CFS is a severe neurological disease that can have a significant impact on everyday school or work life and significantly reduce the quality of life of those who are infected. Moreover, a previous IM is known to be a risk factor for developing multiple sclerosis and Hodgkin lymphoma (12, 13). Although some primary immunodeficiencies are known to be predisposing factors in some cases of fulminant IM (14), the underlying cause of severe or protracted disease is still largely unknown.

Since no direct therapy against IM is known, it is critical to understand the immunological processes during IM. It is known that immune dysregulation, particularly the magnitude of the over-reacting T-cell response, influences the severity of clinical symptoms (15). For this reason, a better knowledge of the cell populations involved in shaping the immune response during IM is one of the prerequisites for better characterizing an effective antiviral immune response and identifying corresponding cellular risk factors for early or late complications of IM.

Myeloid-derived suppressor cells (MDSC) are a heterogeneous group of regulatory immune cells from the myeloid lineage with immunosuppressive activities (16). They can be divided into three distinct subpopulations consisting of early-stage (e-), monocytic (M-), and polymorphonuclear (PMN-) MDSC (17). M-MDSC and PMN-MDSC can develop either from e-MDSC as an immature precursor (18, 19) or from mature monocytes (18, 20–22) or neutrophils (21, 23, 24). Additionally, MDSC are directly released from the bone marrow through emergency myelopoiesis (25–27). MDSC can suppress the immune response of various cells, such as T cells, natural killer cells, or dendritic cells (22, 28), playing an essential role in physiological and pathological conditions (24). Numerous publications have defined a role for MDSC in tumors (29), autoimmune diseases (30), and the immune response in the context of infectious diseases (31–33). It was shown that an increase in MDSC contributes to the chronification of contagious diseases such as hepatitis B (32). Especially during the SARS-CoV-2 pandemic, new insights into MDSC in acute diseases were gained. Various authors report an increase in MDSC in patients with SARS-CoV-2 infection and suspected a connection between this increase and the severity of the clinical course (33). However, the role of MDSC in EBV-associated diseases remains elusive. It has been reported that an increase in MDSC was detected in patients with EBV-associated nasopharyngeal carcinoma (34) and chronically active EBV infection (1, 33). Currently, nothing is known about MDSC in IM. However, a reduction of regulatory T cells (Treg) – another regulatory cell group – is assumed to contribute to the over-reacting T-cell response in primary infection with EBV, contributing to the clinical manifestation of IM (35). In acute viral infections, expansion of phenotypically defined MDSC populations has been interpreted as part of a counter-regulatory response that may limit immunopathology but could also attenuate antiviral effector functions. Accordingly, in IM, associations between MDSC dynamics and organ involvement or inflammatory markers could reflect (i) a regulatory response to inflammation, (ii) a contributing factor to clinical manifestations, or (iii) parallel downstream effects of upstream drivers such as viral burden and the overall inflammatory milieu.

To the best of our knowledge, this is the first prospective clinical-translational study to longitudinally characterize phenotypically defined MDSC-like (sub)populations in pediatric IM. We aimed to describe temporal dynamics of PMN-like, M-like, and e-like MDSC and to assess their associations with contemporaneous clinical, laboratory, and virological features across disease stages. We emphasize that these analyses address phenotypic correlations, not establish causality and address prognostic utility for later outcomes.

## 2 Materials and methods

### 2.1 Study population

The prospective study population is part of the IMMUC study (ClinicalTrials.gov - NCT06002802). A detailed description of the IMMUC study can be found in Bodenhausen et al. (36). Briefly, 200 patients with IM following confirmed acute primary EBV infection were included and investigated at three study visits: V1 as early as possible after symptom onset (T_onset_) but no later than 28 days after onset (DAO) of IM (i.e., DAO 0 = T_onset_), V2 four to six weeks after T_onset_, and V3 six to 12 months after T_onset_. Extensive clinical, laboratory, virological, and serological features were assessed at each visit. Further, the IMMUC scores for IM complexity and IM severity were determined at each visit (36). Briefly, the IM complexity counts the number of 15 pre-defined clinical (IM clinical complexity) and eight pre-defined laboratory (IM laboratory complexity) IM-related features. The IM severity grades the severity of each clinical (IM clinical severity) and laboratory (IM laboratory severity) IM-related feature from S0 to S5. A detailed description of the scoring system can be found in Bodenhausen et al. (36). Additionally, the IM protraction, defined as the presence of any IM-related symptom at V3, and chronic fatigue, defined as the presence of fatigue at V3, were evaluated. Thirty-seven patients described here were investigated for MDSC subpopulations at each visit.

### 2.2 Isolation of peripheral blood mononuclear cells

At each visit, peripheral heparin-anticoagulated whole blood was sampled. Within 4 h, human peripheral blood mononuclear cells (PBMC) were separated by Ficoll density gradient (human Pancoll, PAN-Biotech, Aidenbach, Germany) as described previously (37). PBMC were washed twice using RPMI1640 medium supplemented with 1% Penicillin/Streptomycin and 10% heat-inactivated fetal calf serum (FCS, all from Gibco by Life Technologies, Darmstadt, Germany) (abbr. RPMI-10) and counted by an automated cell counter (ViCell XR 2.04, Beckman Coulter Inc., Krefeld, Germany).

### 2.3 Flow cytometry-based analysis of myeloid-derived suppressor cells

For analysis of MDSC-like cells 1 x 10^6^ freshly isolated PBMC were resuspended in a concentration of 1 x 10^6^ PBMC/100 µL FACS buffer (eBioscience, Invitrogen by Thermo Fisher Scientific, Life Technologies Corp., Carlsbad, USA), filtered using a 100 µm cell strainer (Life Sciences, One Becton Circle, Durham, USA) and stained with a total volume of 11.5 µL antibody mix (**Supplementary Table S1**) for 30 min on ice in the dark. A fluorescence minus one (FMO) control for HLA-DR was also required for the subsequent gating of MDSC-like cells. The antibody mix for the FMO did not contain HLA-DR AL700, but in addition, the corresponding volume of FACS buffer. After surface staining, PBMC were washed twice with 200 µL FACS buffer and resuspended in 300 µL FACS buffer. For Live-Dead staining, a Hoechst 33342 (Biomol GmbH, Hamburg, Germany) dilution of 1:100 was prepared, of which 6 µL was added to each cell sample shortly before measurement on the flow cytometer. Compensation probes were processed identically using 25 µL Ultracomp eBeads Compensation Beads (Invitrogen by Thermo Fisher Scientific, Life Technologies Corp., Carlsbad, USA).

Data were acquired within 4 h after having completed the surface staining of MDSC-like cells with a BD LSRFortessa flow cytometer (Becton, Dickinson and Company, BD Biosciences, San Jose, USA) using BD FACSDiva v6.1.3 software (BD Biosciences, San Jose, USA). PMT voltages were adjusted for all parameters measured using an unstained sample. The analysis was performed using the software FlowJo (FlowJo LLC, Ashland, Oregon, USA; Software version 10).

### 2.4 Gating strategy for MDSC

An exemplary gating strategy for MDSC-like cells is shown in the **Supplementary Information** (**Supplementary Figure S1**). In principle, after gating on single living PBMC, CD33^+^ PBMC were gated. Next, HLA-DR-negative and HLA-DR-negative/low cells were discriminated using a FMO control for HLA-DR. HLA-DR negative cells were defined as events falling within the negative range defined by the FMO. HLA-DR negative/low cells were gated as events falling within the negative range defined by the FMO and just to the right of it. Subsequently, CD14- and CD15-negative e-like MDSC were gated within the HLA-DR-negative cell population. M-like MDSC (CD14^+^CD15^-^) and PMN-like MDSC (CD14^-^CD15^+^) were gated within the HLA-DR-negative/low cell population. Quality control of the gating was performed independently throughout the analysis of each sample.

### 2.5 Statistical analysis

#### 2.5.1 Regression models

**Figure 1:**
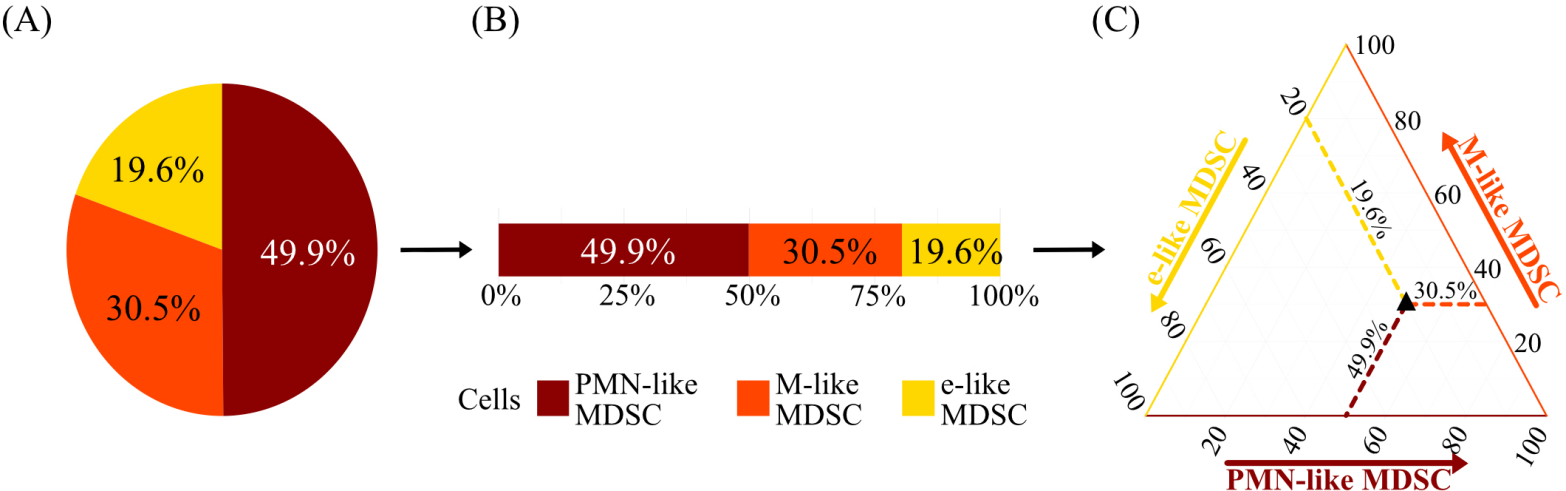
Explanatory plot illustrating how compositional data can be visualized. For each individual and each visit, the total MDSC-like population comprises three subpopulations: PMN-like MDSC, M-like MDSC, and e-like MDSC. Each measurement can be presented as a pie chart **(A)** or a bar chart **(B)**. In the ternary plot **(C)**, the distribution of all compositional data can be visualized in a single plot. The point in the ternary plot represents the composition of the three MDSC-like subpopulations, i.e., the bottom axis (dark red) shows the proportions of PMN-like MDSC, the right axis (orange) shows the proportions of M-like MDSC, and the left axis (yellow) shows the proportions of e-like MDSC.

Linear regression models were used to estimate the effect of clinical and laboratory IM features on the percentage of MDSC-like cells within CD33+ myeloid cells. Dirichlet regressions were used to model the composition of MDSC-like subpopulations (PMN-like MDSC, M-like MDSC, and e-like MDSC), taking into account that their proportions sum to 100% (38), thus providing a more accurate and meaningful analysis than modeling each subpopulation independently. **Figure 1** illustrates how compositional data can be effectively visualized using ternary plots. The results of the Dirichlet regression models were visualized likewise (39). For both the linear and the Dirichlet regression models, medical history, clinical, laboratory, virological, and serological features were included as independent/predictor variables. A separate model was estimated for each feature. Additionally, DAO was included as an independent variable in each model to account for the time dependence of the measurements. All models were further adjusted for sex and age.

#### 2.5.2 Bootstrap likelihood ratio test

A nonparametric bootstrap likelihood ratio test (LRT) was implemented to assess whether adding a feature significantly improved the model fit of both types of regression models compared to a null model that did not include the feature of interest. The observed likelihood ratio (LR_observed_) was computed. Residuals were calculated as the difference between the observed data and the null model’s predictions. B = 5000 bootstrap observations were generated by block-wise sampling per individual to preserve the within-individual correlation structure induced by repeated measurements. These residuals were added to the null predictions to generate bootstrap observations, for which the null and alternative models were refitted. Lastly, the LRT statistics were computed for each bootstrap sample by calculating the log-likelihoods of the null and alternative models and then determining their difference (LR_boot_). The empirical distribution of the LRT statistic and the empirical P-value were constructed from the bootstrap samples (40). If the empirical P-value was below 0.05, the null hypothesis was rejected, indicating a significant improvement.

For the Dirichlet regression, an additional centered log-ratio (CLR) transformation before residual calculation was applied to preserve the compositional structure of the dependent variable (38). Residuals and predictions were sampled and combined in CLR space and subsequently back-transformed. This bootstrap LRT approach allows for a robust assessment of the significance of the added feature by accounting for the complex dependency structure inherent in repeated measures, making it suitable for both compositional and non-compositional data (40, 41). For continuous laboratory variables, values at the lower and upper limits of the pediatric reference range for the mean age were used for predictions (42). For categorical IM features, the relevant categories were included. Predictions for the null model were generated over a DAO range of 1 to 214, using mean age and mode sex. For each statistically significant alternative model, each significant IM feature was separately included in the null model to assess its individual impact.

#### 2.5.3 Temporal and concurrent analysis using mixed-effect models

To investigate whether MDSC-like subpopulation proportions measured at earlier visits (V1 and V2) impacted IM outcomes at V3, LRT with mixed effects models were performed. Outcomes included other airway symptoms, and neurological symptoms at V3, chronic fatigue, and IM protraction. To avoid perfect collinearity inherent in composition data, e-like MDSC was excluded. Additionally, DAO, age, and sex were included as described above. Moreover, associations between MDSC-likesubpopulations measured at V3 and other airway symptoms, neurological symptoms at V3, chronic fatigue, and IM protraction were tested.

#### 2.5.4 Exploratory mixed-effects models of myeloid cells and MDSC-like subsets

To explore whether changes in MDSC-like subset frequencies could reflect phenotypic shifts of mature myeloid populations, additional mixed-effects models were performed to assess associations between monocyte relative count and M-like MDSC, as well as between neutrophils relative count and PMN-like MDSC. For each analysis, the percentage of the respective MDSC-like subset within CD33^+^ myeloid cells was modeled as the dependent variable, with the corresponding myeloid population (monocytes or neutrophils, respectively) included as predictor. All models included DAO, age, and sex as fixed effects and a random intercept to account for repeated measurements.

#### 2.5.5 Other analyses and statistical software

Friedman’s test and Nemenyi’s *post hoc* comparisons were used to analyze the relative frequencies of total MDSC-like cells and their subpopulations (PMN-like MDSC, M-like MDSC, and e-like MDSC) within CD33+ myeloid cells and the proportion of each MDSC-like subpopulation within the total MDSC-like cells over the visits. All hypotheses were tested two-sided with a significance level of α = 0.05. P-values are indicated as follows: P < 0.1., P < 0.05*, P < 0.01**, and P < 0.001***.

Analyses were performed using R, version 4.3.3 “Angel Food Cake” (The R Foundation for Statistical Computing, Vienna, Austria). The results of the Dirichlet regression models were estimated likewise (39). Ternary plots were generated using the ggtern (43). The R code for the bootstrap procedure is openly available on github.com/LangedeLuna/IMMUC_MDSC.

### 2.6 Study approval

The study was approved by the institutional review board of the Technical University of Munich (ref. 112/14) and adheres to the Declaration of Helsinki and its later amendments. Written informed consent was obtained from the participants and from their legal guardians prior to inclusion.

## 3 Results

### 3.1 Patient baseline characteristics

Patient baseline characteristics are shown in **Table 1**. The study included a total of 37 patients: 19/37 (51.35%) children (2–11 years) and 18/37 (48.65%) adolescents (12–17 years). 21/37 (56.76%) patients were female, and 16/37 (43.24%) were male. The mean age of the patients was 10.84 ± 4.75 years (range: 2 to 17). 35/37 (94.59%) and 36/37 (97.30%) patients were within normal body height and BMI ranges. Further baseline medical history characteristics (**Supplementary Table S2**), clinical and laboratory IM features (**Supplementary Tables S3**, **S4**), and virological IM features (**Supplementary Table S5**) are shown in the **Supplementary Material**.

**Table 1:** Baseline characteristics at study inclusion.

Patient characteristics	Proportion
**Sex**	**Number/Total (%)**
Female	21/37 (56.8%)
Male	16/37 (43.2%)
**Age groups**	**Number/Total (%)**
Children 2–11 years	19/37 (51.4%)
Adolescents 12–17 years	18/37 (48.7%)
**Body height^1^**	**Number/Total (%)**
Below normal range	1/37 (2.7%)
Within normal range	35/37 (94.6%)
Above normal range	1/37 (2.7%)
**Body mass index (BMI)^1^**	**Number/Total (%)**
Below normal range	1/37 (2.7%)
Within normal range	36/37 (97.3%)
Above normal range	0/37 (0.0%)

^1^Age-adapted normal range of body measures (3^rd^ – 97^th^ percentile) (44, 45).

### 3.2 Comparable frequencies of total MDSC-like cells during the early and late phases of IM

**Figure 2:**
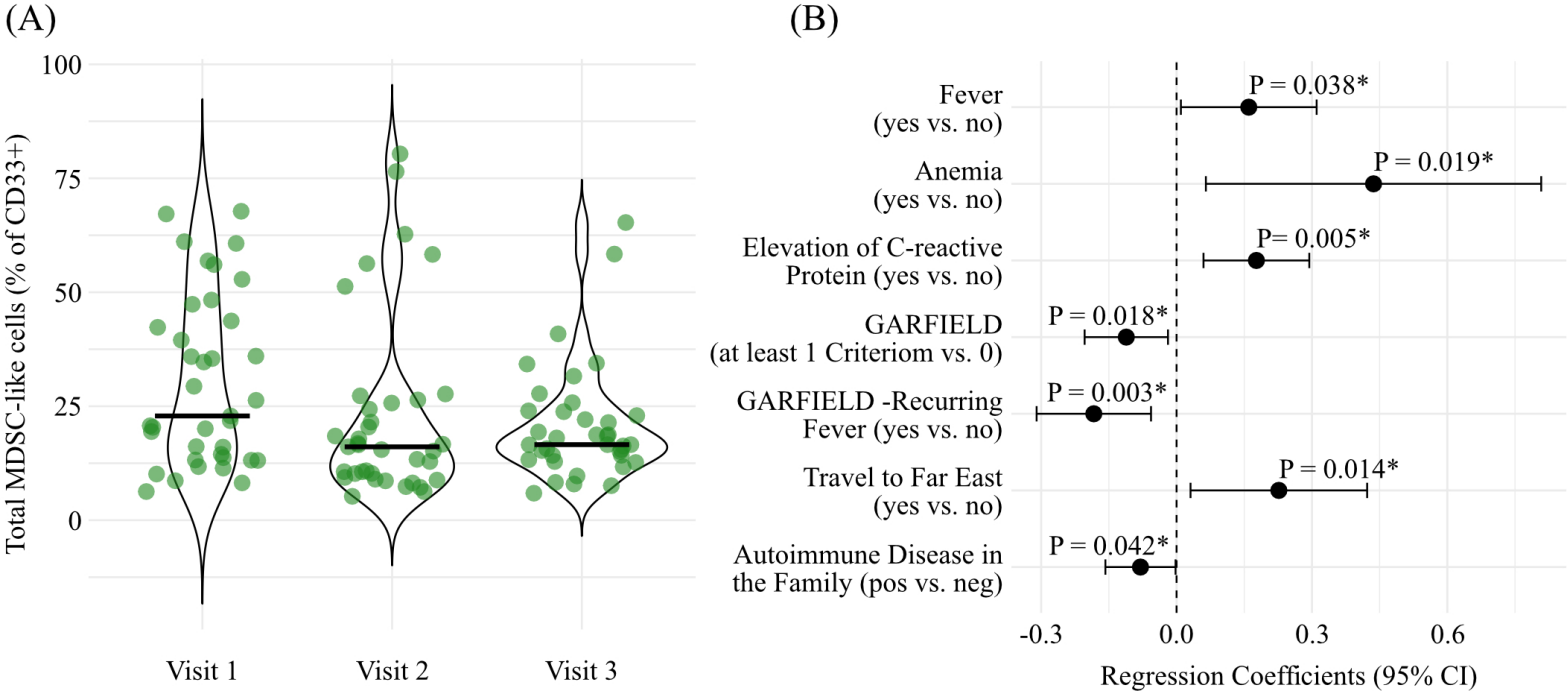
The relative frequency of total MDSC-like cells. The violin plots depict the proportion of total MDSC in % of CD33+ myeloid cells **(A)** across the three study visits. The horizontal lines mark the median. The forest plot **(B)** shows the mean standardized regression coefficients and their 95%-CI for each feature significantly influencing the total MDSC-like population. The statistical significance was tested using the Bootstrap Likelihood Ratio Test (LR_boot_). The empirical P-values are shown above each mean regression coefficient.

First, we examined the relative frequency of total MDSC-like cells across the three visits. Here, we observed no significant differences in frequencies of MDSC-like cells between V1 (median: 22.87%, IQR: 13.73% to 43.71%), V2 (median: 16.10%, IQR: 10.23% to 25.70%), and V3 (median: 16.58%, IQR: 14.22% to 23.81%) (*P* = 0.182) (**Figure 2A**). However, LRT_boot_ revealed that the frequencies of total MDSC-like cells were significantly influenced by markers of systemic inflammation, including the presence of fever (*P* = 0.038), anemia (*P* = 0.019), and elevated C-reactive protein levels (*P* = 0.005). In addition, clinical warning signs of immune dysregulation were associated with frequencies of total MDSC-like cells, namely the presence of at least one GARFIELD criterion (*P* = 0.018) and recurrent fever as defined by the GARFIELD framework (*P* = 0.003). Finally, established risk-related factors, including a history of travel to the Far East (*P* = 0.014) and a family history of autoimmune disease (*P* = 0.042), were also identified as significant contributors (**Figure 2B**).

### 3.3 Temporal dynamics of PMN-like, M-like, and e-like MDSC during the early and late phases of IM

**Figure 3:**
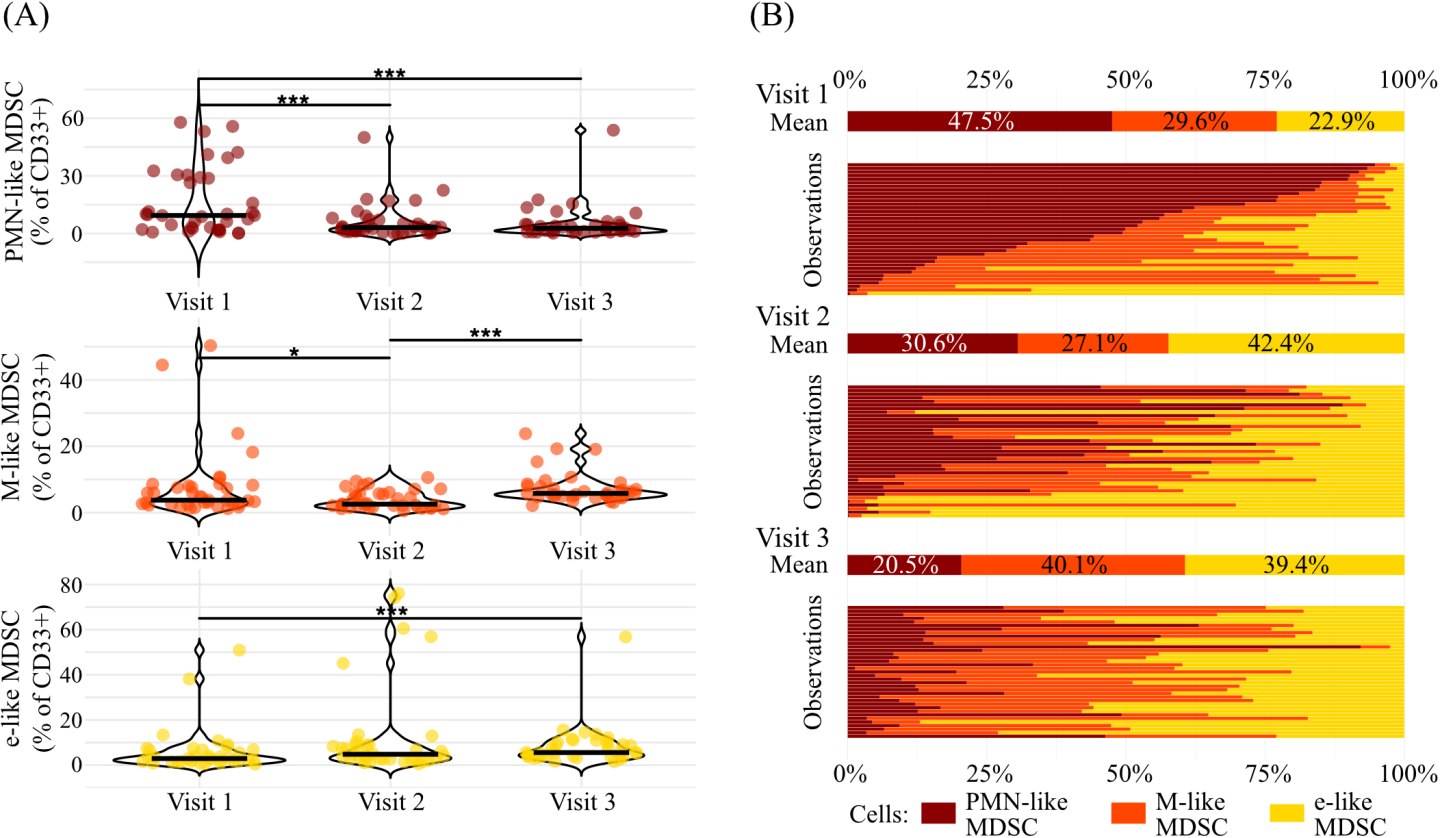
The distribution of PMN-like, M-like, and e-like MDSC across the three study visits. The violin plots **(A)** depict the percentage distribution of PMN-like MDSC (red), M-like MDSC (orange), and e-like MDSC (yellow) as a proportion of CD33+ myeloid cells across the three study visits. The dots depict each observation, and the horizontal lines mark the median. The statistical significance was tested using Friedman’s test and Nemenyi’s *post hoc* comparisons. The mean and individual observation proportions of MDSC-like subpopulations across three different visits are shown in **(B)**. Each set of bars represents one visit, displaying the percentage distribution of the cell types PMN-like MDSC (red), M-like MDSC (orange), and e-like MDSC (yellow). Bars are ordered consistently across visits. * P < 0.05; *** P < 0.001.

Though the total frequencies of MDSC-like cells does not change over the course of IM, the temporal dynamics of the relative frequencies of PMN-like, M-like, and e-like MDSC subpopulations among CD33+ myeloid cells showed distinct changes throughout the study period (**Figure 3A**). The frequency of PMN-like MDSC decreased significantly from V1 to V2 (*P* < 0.001) and from V1 to V3 (*P* < 0.001). In contrast, the frequency of M-like MDSC decreased from V1 to V2 (*P* = 0.015) and increased from V2 to V3 (*P* < 0.001). Meanwhile, the frequency of e-like MDSC increased from V1 to V3 (*P* < 0.001). These changes in the MDSC-like subpopulations relative to CD33+ myeloid cells are reflected in the observed proportions of the MDSC-like subpopulations relative to the total MDSC-like population, as shown in **Figure 3B**. At V1, PMN-like MDSC accounted for 47.5% of all MDSC-like cells. They significantly decreased to frequencies of 30.6% at V2 (V1 vs. V2, *P* = 0.003) and further to 20.5% at V3 (V1 vs. V3, *P* < 0.001). Concomitantly, the proportion of M-like MDSC remained stable from 29.6% at V1 to 27.1% at V2 (V1 vs. V2, *P* = 0.476), before increasing significantly to 40.1% at V3 (V2 vs. V3, *P* = 0.002). e-like MDSC contributions increased from 22.9% at V1 to 42.4% at V2 (V1 vs. V2, *P* < 0.001) and remained at high level between V2 and V3 (39.4%) (V1 vs. V3, *P* < 0.001).

### 3.4 Exploratory associations between myeloid cells and MDSC-like subpopulations

To explore whether the observed temporal dynamics of MDSC-like subpopulations could reflect phenotypic shifts of mature myeloid cells, associations between relative monocyte count and M-like MDSC, as well as between relative neutrophil count and PMN-like MDSC, were assessed using linear mixed effects regression analyses. No statistically significant associations were observed between relative monocyte count and M-like MDSC (*P* = 0.101), nor between relative neutrophil count and PMN-like MDSC (*P* = 0.198) (**Supplementary Figure 3**).

### 3.5 Dynamics of PMN-like, M-like, and e-like MDSC proportions depending on days after onset

**Figure 4:**
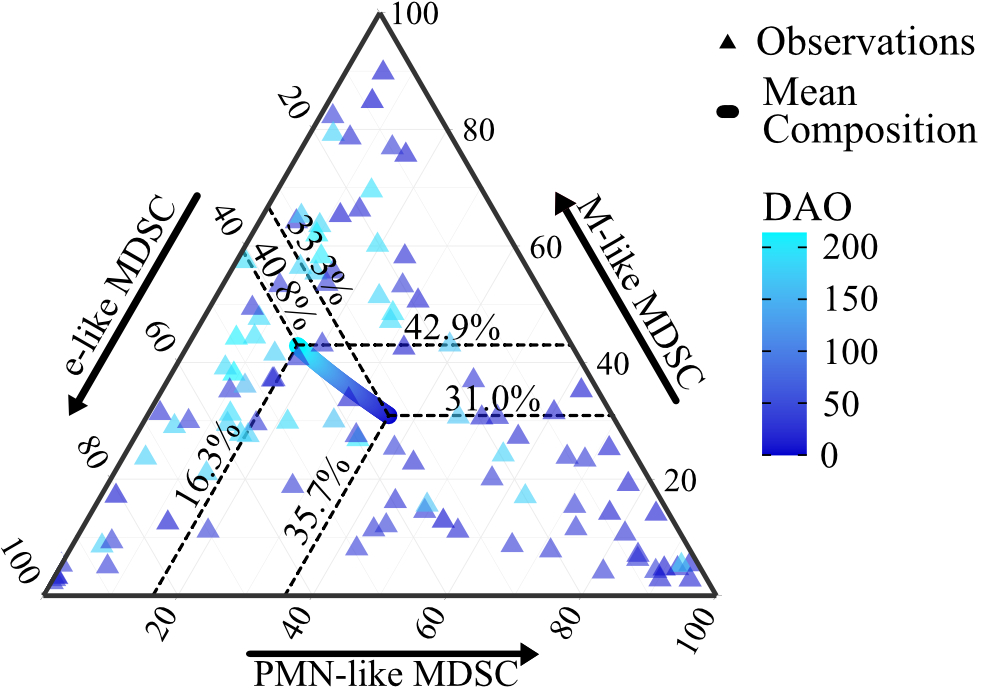
The proportions of e-like MDSC, M-like MDSC, and PMN-like MDSC subpopulations at different days after onset. The ternary plot illustrates the proportions of e-like MDSC, M-like MDSC, and PMN-like MDSC cell subpopulations at different Days After Onset (DAO), with individual observations represented by triangles and the mean composition derived from the Dirichlet regression model shown as a curve. The model included DAO values ranging from 1 to 214 and is adjusted for mean age (10.84 years) and sex mode (female). Dashed lines indicate the relative frequency of each cell subpopulation at baseline (DAO 1) and final time point (DAO 214). Early DAO values are depicted in dark blue, late DAO values are in light blue.

Next, we analyzed the compositional temporal dynamics of the three MDSC-like subpopulations over the study period. At the beginning of the observational window (DAO = 1), the mean composition of the subpopulations comprised 35.7% of PMN-like MDSC, 31.0% of M-like MDSC, and 33.3% of e-like MDSC (**Figure 4**). By the end of the observational window (DAO = 214), notable shifts are observed: the mean proportion of PMN-like MDSC decreased to 16.3%, M-like MDSC increased to 42.9%, and e-like MDSC increased to 40.8%, underscoring the ongoing changes in distribution of MDSC-like cells (**Figure 4**).

### 3.6 Relationship between different IM features and the dynamics of PMN-like, M-like, and e-like MDSC proportions over time

**Figure 5:**
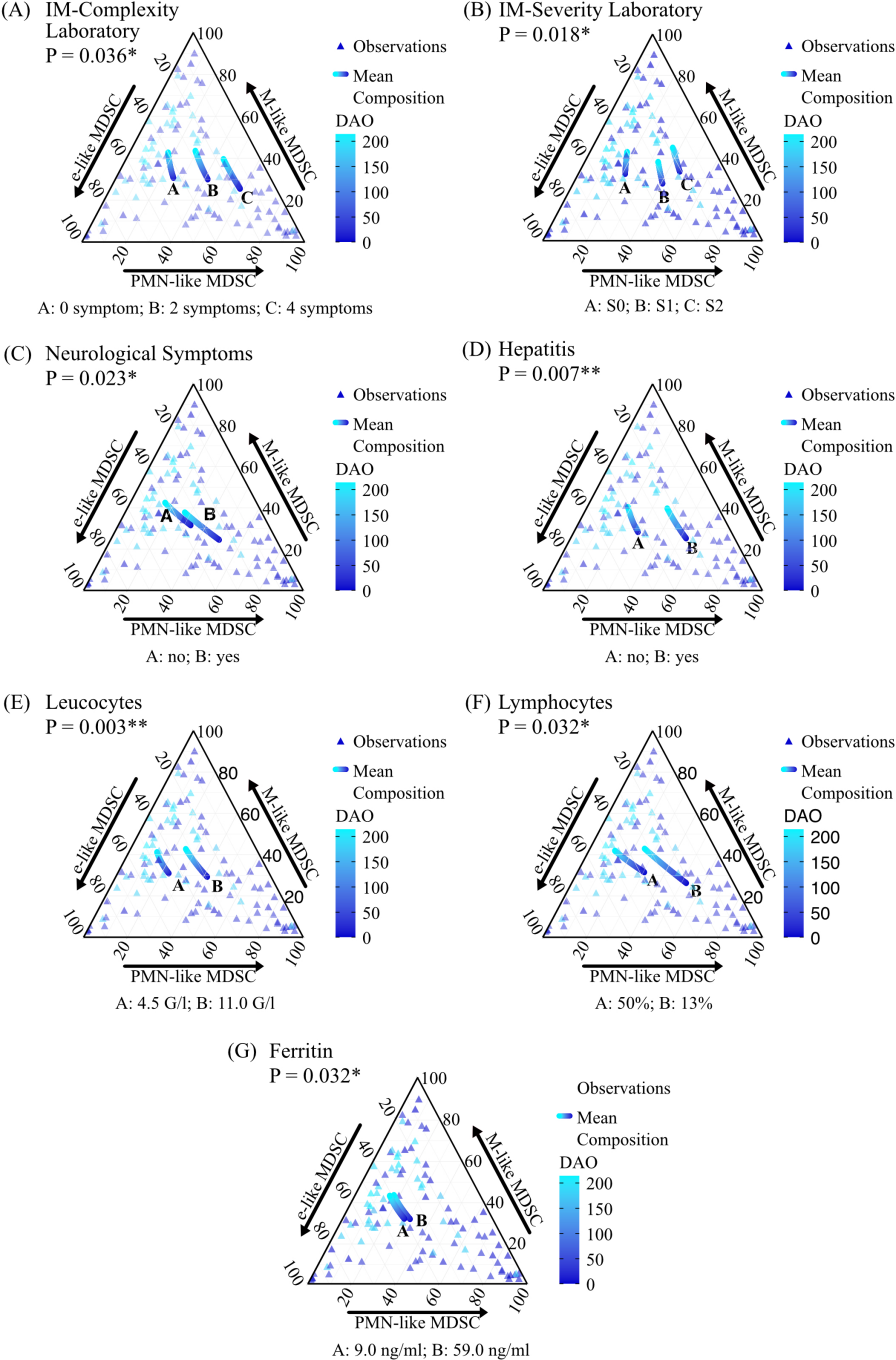
Relationship between different IM features and the dynamics of PMN-like, M-like, and e-like MDSC proportions over time. The ternary plots **(A–G)** illustrate the proportions of PMN-like MDSC, M-like MDSC, and e-like MDSC subpopulations for the statistically significant features identified by the bootstrap likelihood ratio test (LR_boot_). Triangles represent individual observations, and the curves represent the mean composition derived from the Dirichlet regression models. Models included DAO values from 1 to 214, mean age (10.84 years), and sex mode (female). For the significant features the values used were: IM-Complexity laboratory (0, 2 and 4 symptoms), IM-Severity laboratory (S0, S1, and S2), presence of neurological symptoms (no/yes), presence of hepatitis (no/yes), absolute leucocyte count (4.50 G/l, 11.00 G/l), relative lymphocyte count (50% and 13%) and ferritin level (9.00 ng/ml and 59.00 ng/ml). * P < 0.05; ** P < 0.01.

We examined whether a relationship exists between different clinical, laboratory, and virological IM features and the compositional dynamics of the three MDSC-like subpopulations. The variables IM complexity laboratory (*P* = 0.035; **Figure 5A**), IM severity laboratory (*P* = 0.023; **Figure 5B**), presence of neurological symptoms (*P* = 0.025; **Figure 5C**) and/or hepatitis (*P* = 0.007; **Figure 5D**), the absolute leucocyte count (*P* = 0.002; **Figure 5E**), relative lymphocyte count (*P* = 0.036, **Figure 5F**), and ferritin level (*P* = 0.025; **Figure 5G**) significantly influenced the compositional dynamics of the MDSC-like subpopulations.

Specifically, the proportion of PMN-like MDSC increased with the laboratory IM complexity (**Figure 5A**). Similar trends were observed for the laboratory IM severity, absolute leukocyte count, and ferritin level (**Figures 5B, E**, and **G**). The presence of neurological symptoms and hepatitis was also associated with increased PMN-like MDSC proportions (**Figures 5C, D**). Conversely, PMN-like MDSC proportions decreased with increasing relative lymphocyte count. (**Figure 5F**).

M-like MDSC proportions were lower with increasing complexity and increased with extending DAO in all complexity categories. (**Figure 5A**). Regarding the IM severity laboratory, proportions decreased at medium severity and rose with increasing severity (**Figure 5B**). For both absolute leucocyte counts and ferritin levels, M-like MDSC proportions remained stable as these markers increased (**Figures 5E, G**). The presence of neurological symptoms and hepatitis was associated with lower M-like MDSC proportions throughout the course of disease (**Figures 5C, D**). Early in the disease, M-like MDSC proportions increased with increasing relative lymphocyte counts, whereas later in the disease, proportions were similar (**Figure 5F**).

For e-like MDSC, proportions were lower with increasing IM complexity laboratory, IM severity laboratory, absolute leucocyte count, and ferritin level, but were higher with higher relative lymphocyte count (**Figures 5A, B, E–G**). Notably, e-like MDSC proportions were lower in the presence of neurological symptoms or hepatitis (**Figures 5C, D**). The proportions of the three MDSC-like subpopulations are illustrated in **Figures 5A–G**.

### 3.7 Impact of PMN-like and M-like MDSC on long-term IM outcomes

**Figure 6:**
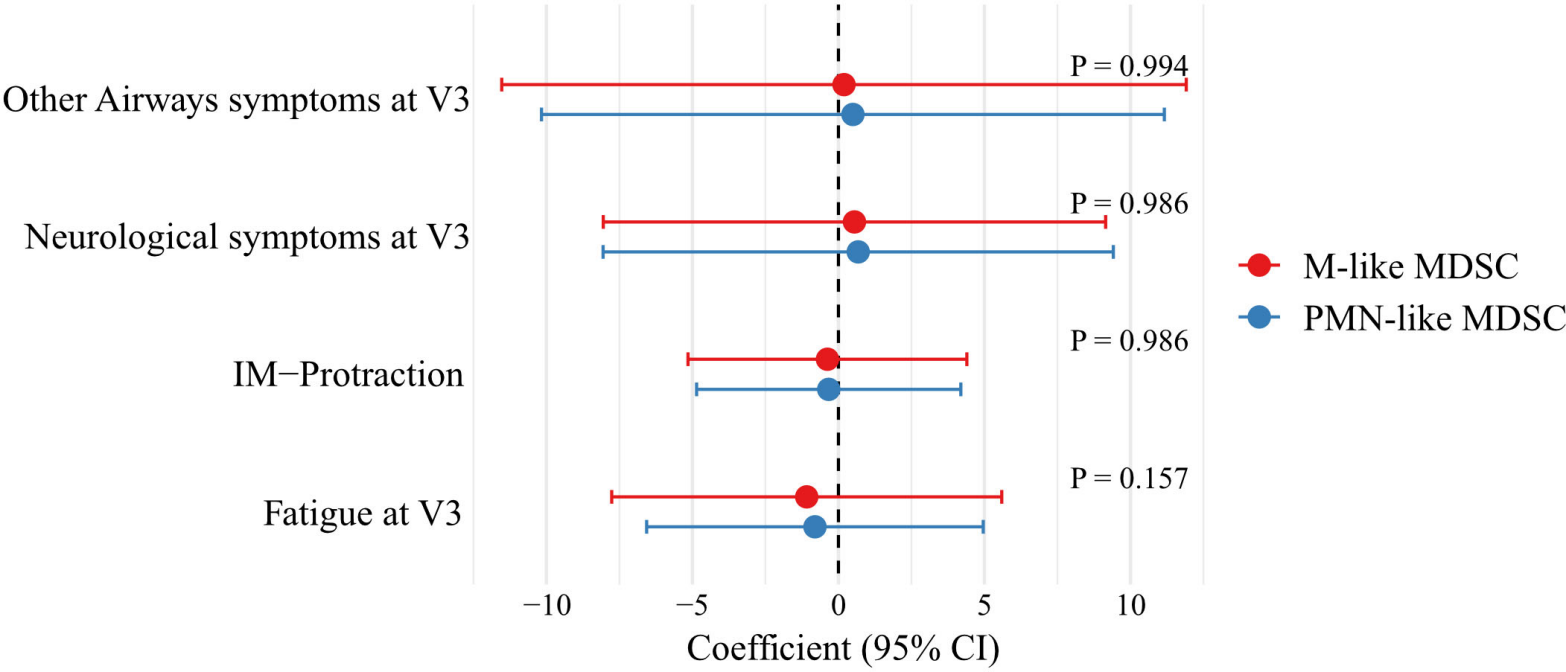
Impact of PMN-like and M-like MDSC on long-term IM outcomes. Forest plot showing the standardized estimated regression coefficients and 95% confidence intervals for the association between PMN-like MDSC and M-like MDSC proportions measured at V1 and V2 and outcomes at V3, including Other Airways symptoms, Neurological symptoms, Fatigue, and IM-Protraction. The coefficients reflect the associations identified through mixed-effects analysis, with statistical significance assessed by the Likelihood Ratio Test (LRT).

Analyzing a potential correlation between long-term clinical outcome (clinical symptoms at V3) showed that earlier PMN-like MDSC and M-like MDSC proportions from V1 and V2 did not significantly influence later clinical outcomes, such as other airway symptoms at V3, neurological symptoms at V3, chronic fatigue, and IM protraction (**Figure 6**). These findings suggest that changes in MDSC-like proportions did not affect later outcomes. Additionally, MDSC-like subpopulations measured at V3 did not show a significant association with other airway symptoms (*P* = 0.981) and neurological symptoms at V3(*P* = 0.902), chronic fatigue (*P* = 0.834), or IM protraction (*P* = 0.852).

### 3.8 Other analyses using linear regression

To assess if the Dirichlet regression is appropriate for modeling the MDSC-like subpopulations, individual linear regression analyses were performed for each subpopulation (PMN-like MDSC, M-like MDSC, and e-like MDSC) as outcomes, using the same independent variables as in the Dirichlet regression model. In the linear regression models, the proportion of each subpopulation was modeled relative to the frequencies of MDSC-like subsets in CD33+ myeloid cells across all time points. The results, which are presented in **Supplementary Material Supplementary Figure 2**, identified significant associations between distinct IM features which were different for each MDSC-like subpopulation: absolute leucocytes count (*P* = 0.002), relative lymphocyte count (*P* = 0.008), ferritin level (*P* = 0.020), diphtheria toxoid titer (*P* = 0.021), asthma (*P* = 0.048), Mc Isaac score (*P* = 0.022), the number of ELVIS signs of primary immunodeficiency (*P* = 0.047), and the ELVIS sign of primary immunodeficiency increased sum of minor infections (*P* = 0.032) were associated with PMN-MDSC; elevation of C-reactive protein (*P* = 0.032), the GARFIELD sign of primary immunodeficiency eczema, including neurodermatitis (*P* = 0.011), positive EBV EA-p138 IgG status (*P* = 0.015), and neurodermatitis in the family (*P* = 0.027) were associated with M-MDSC; and hepatitis (*P* = 0.035), HHV-6 DNA load (*P* = 0.025), travel to Far East within the last 6 months (*P* = 0.009), the ELVIS signs of primary immunodeficiency multiple localization (*P* = 0.031), and relapsing course of infection (*P* = 0.038) were associated with e-like MDSC.

## 4 Discussion

MDSC comprise a heterogeneous population of myeloid regulatory cells found in cancer and infectious diseases (22, 31). MDSC have regulatory functions and can help to resolve inflammation by suppressing the immune response of T cells, NK cells, or dendritic cells (22, 28) and therefore play an essential role in reducing immune-mediated pathology (24, 46). On the other hand, the immunosuppressive function of MDSC on T cells can hinder the establishment of an effective immune response and may prevent a robust immune response to the pathogen (46). Although much is known about the role of MDSC in cancer, autoimmune, and infectious diseases (22, 30, 33, 47, 48), their role in the development and influence on the severity and progression of IM is largely unknown.

To fill this gap, we conducted a clinical translational prospective study unique in its approach, as we conducted longitudinal analyses of the PMN-like, M-like, and e-like MDSC frequencies in pediatric patients during the early and late phases of IM. This novel approach allowed us to provide reliable insights into the relationship between frequencies of MDSC-like cells and the complexity and severity of laboratory, clinical, and virological IM features.

Importantly, given the observational design, the reported relationships represent associations and do not allow inference on the direction of causality. MDSC dynamics may contribute to, result from, or occur in parallel with upstream drivers such as overall inflammation or viral burden.

The proportion of total MDSC-like cells relative to CD33+ myeloid cells did not show a statistically significant change over the course of IM, although median values declined from visit 1 to later visits. Several clinical and immunological features were statistically associated with the frequency of total MDSC-like cells. Positive associations were observed for travel to Asia or the Far East, fever, anemia, and C-reactive protein elevation, whereas autoimmune disease in the family, the GARFIELD warning sign recurrent fever of unknown origin, and the presence of GARFIELD warning signs were associated with lower proportions of total MDSC-like cells. Although anemia showed the largest association among these variables, the overall magnitude of the associations was modest in the context of the observed between-subject variability in proportions of total MDSC–like cells and is therefore unlikely to be clinically meaningful.

One of our most significant findings was the temporal dynamics of the MDSC-like subpopulations PMN-like, M-like, and e-like MDSC during the early and late phases of IM. The analysis revealed that PMN-like MDSC represented the most frequent MDSC-like subpopulation at the early stage of IM (V1), and their frequencies significantly decreased during the later phase of IM (V1 to V3). In contrast, M-like and e-like MDSC frequencies increased during the course of IM (V1 to V3). Based on the current understanding of the development of the various MDSC–like subpopulations, these different courses can be explained as follows: The initial increase in PMN-like MDSC frequencies may be primarily due to the reprogramming of mature neutrophils which are activated quickly in the course of infections but only have a short lifespan (24, 49–53). In contrast, emergency myelopoiesis (25, 54–57) and reprogramming of monocytes to M-MDSC (27) occur with a time delay and monocytes have a longer lifespan, which could explain the later and sustained increase of e-like and M-like MDSC. Importantly, exploratory analyses did not provide evidence for associations between relative monocyte/neutrophil counts and the corresponding MDSC-like subpopulations, suggesting that the observed MDSC-like dynamics are not simply a proxy for routine differential count shifts. Similar courses were found in patients with sepsis (27, 58). However, the frequencies of MDSC-like subpopulations were not associated with IM severity or IM complexity. In turn, the proportional composition of the MDSC-like subpopulations was significantly associated with IM complexity and severity. This is in line with previously mentioned studies regarding COVID-19 outcome (48, 59–61). There, significantly higher frequencies of PMN-like MDSC were observed in the non-survivor compared with the survivor group, which led to the suggestion that PMN-like MDSC percentages could be predictive of COVID-19 disease outcome. Bline et al. also observed increased frequencies of PMN-like MDSC in hospitalized COVID-19 pediatric patients compared to healthy children (62). Our finding of higher proportions of PMN-like MDSC with the higher complexity and severity of laboratory IM features is compatible with the hypothesis that higher PMN-MDSC proportions may be associated with a reduced T-cell-driven immune response. This is in line with the report of Agrati et al. (48), who observed a substantial increase in PMN-MDSC frequencies in patients with severe COVID-19, accompanied by the suppression of T-cell proliferation and cytokine production. Reports showing that PMN-MDSC have superior suppressive capacity among MDSC underline this assumption (63, 64). However, it remains unclear whether the increase in PMN-MDSC frequencies is a mechanism for controlling the inflammatory response induced by EBV infection or whether, conversely, disease-related factors induce the expansion and activation of MDSC to exacerbate inflammation, or whether both are driven by upstream factors such as systemic inflammation and/or viral burden. Both the protective and pathogenic roles of MDSC are described in different types of biological processes and disease settings (31, 48, 65). Notably, we did not observe predictive associations between early MDSC-like subpopulation proportions and later clinical outcomes at V3, suggesting that MDSC-like dynamics in IM primarily reflect contemporaneous immune regulation during active disease phases rather than serving as prognostic biomarkers.

The observed correlation of PMN-like MDSC levels with increased absolute leucocyte counts at the respective visits could be a hint that cytokines secreted by leucocytes, e.g., IFN-γ, IL-4, IL-13, IL-1ß, and TGF-ß, may activate MDSC and are involved in regulating PMN-MDSC frequencies (66).

It is worth mentioning that publications often do not differentiate between the various subpopulations of MDSC, which has led to limited knowledge about the individual subpopulations in the field of infectious virology. Our data suggests that it could be essential to pay more attention to changes in MDSC composition during the course of viral infection. Our finding of consistent frequencies of MDSC-like cells throughout the course of IM across individual study visits underscores this point. It should also be noted that it is extremely important that MDSC be freshly analyzed within four hours of collection (67). Studies have shown that freezing in particular decreases the yield of M-MDSC (68).

Given the results of this study, it would be informative to investigate PMN-like MDSC frequencies longitudinally in a larger cohort of pediatric IM patients to further pursue a potential therapeutic approach in targeting this MDSC subpopulation. Of particular interest would be to determine the (PMN-) MDSC frequencies before the onset of IM to learn whether the MDSC subpopulation frequencies return to their baseline levels after IM. However, to evaluate this, a prospective study would be necessary in which the baseline values of the MDSC frequencies are also recorded. In addition, there is little knowledge about the typical values of MDSC subpopulations in pediatric cohorts. Future studies should therefore include a control cohort of healthy children to gain new insights into the corresponding reference values. A study on healthy children involving one or more blood drawings, however, faces ethical hurdles.

There are limitations to our study. Since phenotypic markers unique to MDSC have not been identified so far, their immunosuppressive function on T cells must be demonstrated by additional means such as co-culture experiments (22). Due to limitations in blood volumes in this cohort of pediatric IM patients, we could not conduct those experiments. However, this does not allow for a definitive distinction between M-MDSC or PMN-MDSC and activated monocytes or neutrophils. What suggests that it is M-MDSC and not activated monocytes is that activated monocytes, unlike M-MDSC, typically exhibit upregulation of HLA-DR, which was also shown in a pediatric cohort with IM (69–71). A phenotypic distinction between PMN-MDSC and activated neutrophils without functional evidence of immunosuppressive capacity is even more difficult. Meanwhile, additional markers have been identified, such as LOX-1 (23, 72) or CD84 and CD52 (73), which could enable a better distinction between PMN-MDSC and activated neutrophils in subsequent studies. However, these markers have primarily been researched in cancer. Further studies are necessary to validate the significance of these additional markers as suitable markers for PMN-MDSC in infectious diseases. The specificity of LOX-1 as a marker for PMN-MDSC in infectious diseases may be reduced by the fact that LOX-1 expression is induced by ER stress, which could also lead to transient LOX-1 expression on activated neutrophils (23, 74). CD52 and CD84 are also not specific to PMN-MDSC (75–78). What methodically suggests that it is PMN-MDSC and not activated neutrophils is that PMN-MDSC are found as low-density neutrophils in the PBMC fraction, while classical neutrophils are found in the dense granulocyte fraction (23, 79, 80). However, there is meanwhile evidence that some neutrophils can migrate into the PBMC layer after activation (81, 82). These low-density neutrophils appear to be a heterogeneous group of cells that are not equivalent to PMN-MDSC and also exhibit some pro-inflammatory effects (83). Therefore, it would be very useful and important for future studies to supplement additional markers and/or functional assays to clearly distinguish PMN-MDSC from activated neutrophils. However, since we limit ourselves in this study to a definition of MDSC based on surface markers, we have used the following terminology in the paper to avoid misleading statements: “MDSC-like cells,” “PMN-like,” “M-like,” and “e-like” MDSC.

In summary, we report significant temporal dynamics of the MDSC-like subpopulations PMN-like, M-like, and e-like MDSC during the early and late phases of IM. This discovery revealed that PMN-like MDSC represented the most frequent MDSC-like subpopulation at the early stage of IM, and the proportion of PMN-like MDSC increased with the IM complexity and the severity laboratory score.

Our data highlight the importance of in-depth analyses of the MDSC subpopulations in further studies of IM to obtain a more differentiated picture of the ongoing changes in MDSC distribution over the course of IM. Future studies should also include functional suppression assays and consider the use of additional markers for more definitive phenotyping of MDSC.

## Data Availability

The raw data supporting the conclusions of this article will be made available by the authors, without undue reservation.
