# Supplementary material for: The composition of MDSC-subpopulations PMN-like, M-like, and e-like MDSC is associated with the severity of infectious mononucleosis in pediatric patients
Source: Front Immunol. 2026 Mar 30;17:1729699. doi: 10.3389/fimmu.2026.1729699 (PMC13071033; doi:10.3389/fimmu.2026.1729699)
Supplement: Supplementary file 1 [file Supplementaryfile1.pdf]

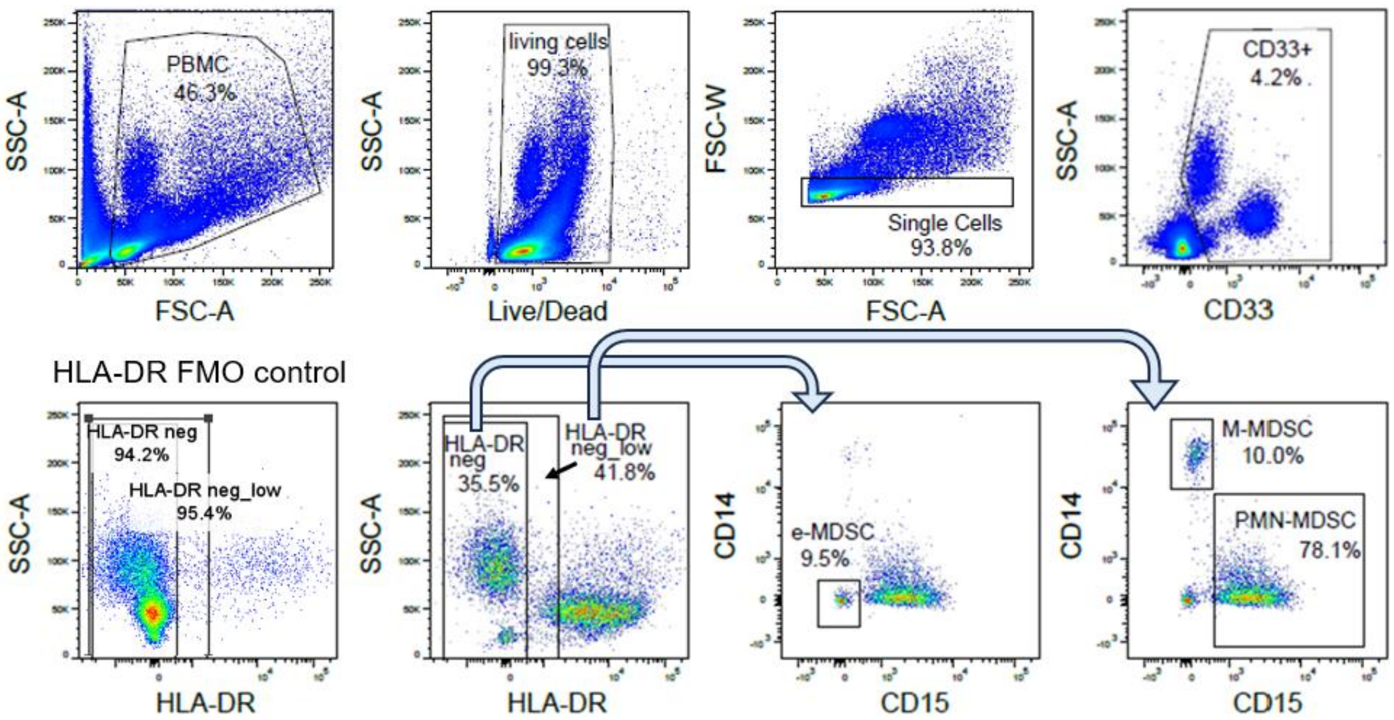

SUPPLEMENTARY FIGURE S1. MDSC gating strategy. Flow cytometry plots showing the gating strategy applied to determine the three MDSC subpopulations. In detail, after gating on single living PBMC, $CD33^+$ PBMC were gated. Next, HLA-DR-negative and HLA-DR-negative/low cells were differentiated by defining the gates for both populations using the FMO control. Subsequently, CD14- and CD15-negative e-like MDSC were gated within the HLA-DR-negative cell population. M-like MDSC ($CD14^+CD15^-$) and PMN-like MDSC ($CD14^-CD15^+$) were gated within the HLA-DR-negative/low cell population. Arrows between plots indicate subgating.
