# Supplementary material for: The composition of MDSC-subpopulations PMN-like, M-like, and e-like MDSC is associated with the severity of infectious mononucleosis in pediatric patients
Source: Front Immunol. 2026 Mar 30;17:1729699. doi: 10.3389/fimmu.2026.1729699 (PMC13071033; doi:10.3389/fimmu.2026.1729699)
Supplement: Supplementary file 2 [file Supplementaryfile2.pdf]

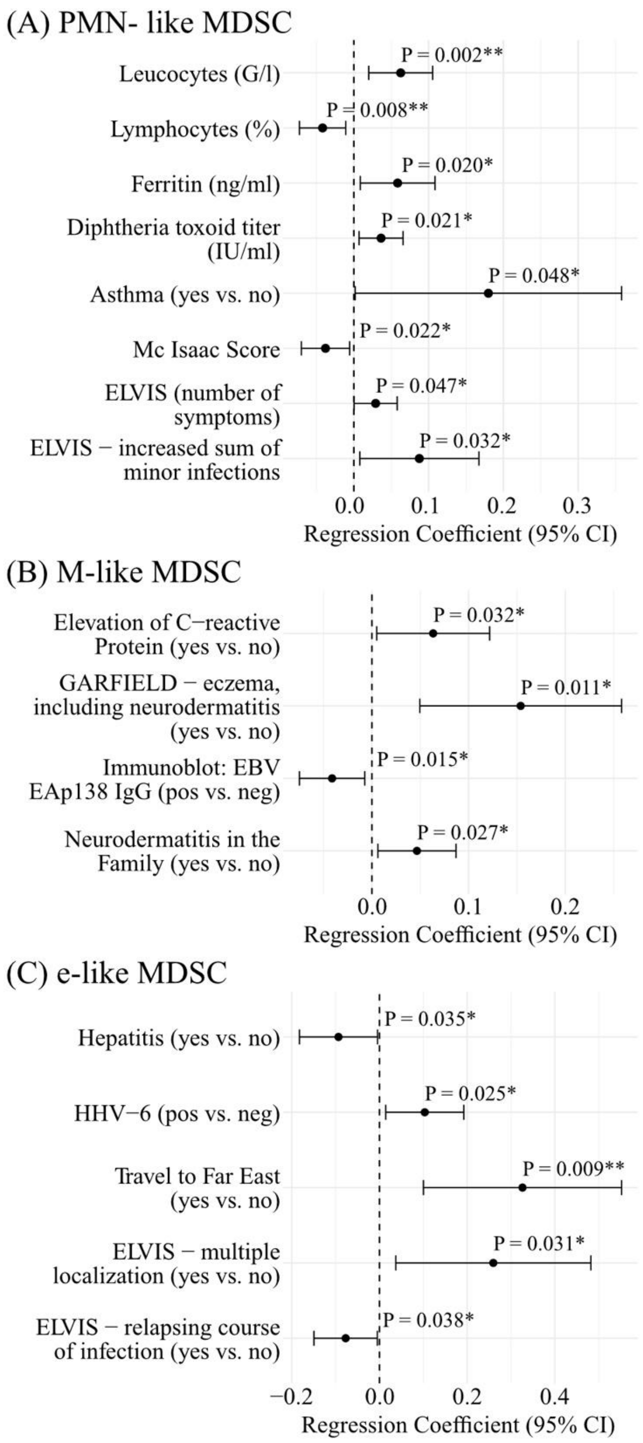

SUPPLEMENTARY FIGURE S2. The forest plots show the standardized regression coefficients and 95%-CI for features that significantly influenced the PMN-like MDSC (A), M-like MDSC (B), and e-like MDSC (C) subpopulations as a proportion of CD33+ myeloid cells. The statistical significance was tested using the Bootstrap Likelihood Ratio Test (LR_{boot}). The empirical P-values are shown above each mean regression coefficient.
