# Supplementary material for: The composition of MDSC-subpopulations PMN-like, M-like, and e-like MDSC is associated with the severity of infectious mononucleosis in pediatric patients
Source: Front Immunol. 2026 Mar 30;17:1729699. doi: 10.3389/fimmu.2026.1729699 (PMC13071033; doi:10.3389/fimmu.2026.1729699)
Supplement: Supplementary file 3 [file Supplementaryfile3.pdf]

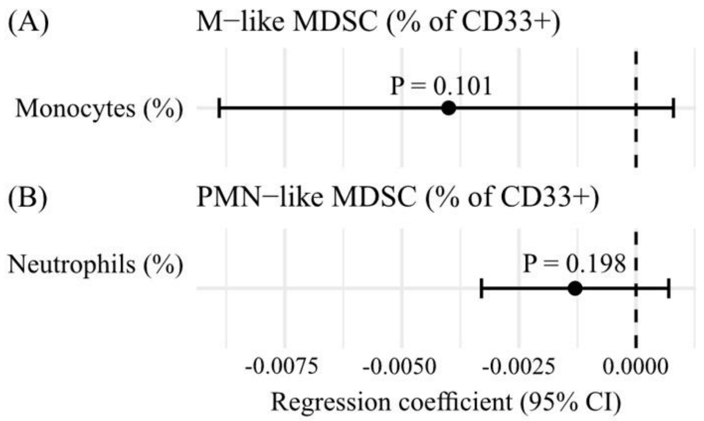

SUPPLEMENTARY FIGURE S3. The forest plots display the estimated regression coefficients and 95%-CI derived from linear mixed-effect models. Panel (A) shows the association between the relative monocyte count and M-like MDSC expressed as a proportion of CD33+ myeloid cells. Panel (B) shows the association between the relative neutrophil count and PMN-like MDSC expressed as a proportion of CD33+ myeloid cells. P-values are shown above each regression coefficient.
