# Supplementary material for: The composition of MDSC-subpopulations PMN-like, M-like, and e-like MDSC is associated with the severity of infectious mononucleosis in pediatric patients
Source: Front Immunol. 2026 Mar 30;17:1729699. doi: 10.3389/fimmu.2026.1729699 (PMC13071033; doi:10.3389/fimmu.2026.1729699)
Supplement: Supplementary file 4 [file Supplementaryfile4.pdf]

SUPPLEMENTARY TABLES

SUPPLEMENTARY TABLE S1. Antibodies for MDSC staining.

Antibody	Fluorochrome	Vendor	Clone	Concentration [µg/mL]
CD15	PerCP-Cy5.5	BioLegend	W6D3	0.91
CD14	PE-Cy7	eBioscience	61D3	0.91
CD33	eF450	eBioscience	P67.6	1.14
HLA-DR	Al700	eBioscience	LN3	0.03
Hoechst 33342	-	Biomol GmbH	-	0.04

SUPPLEMENTARY TABLE S2. Baseline Patient Characteristics – General Medical History.

**Vaccination status**	**Number / Total (%)**
Complete vaccination status[1]	31/37 (83.8%)
Incomplete vaccination status[1]	6/37 (16.2%)
**Diphtheria toxoid titer[2]**	**Number / Total (%)**
Below normal range	0/36 (0.0%)
Within normal range	36/36 (100.0%)
**Tetanus toxoid titer[2]**	**Number / Total (%)**
Below normal range	1/37 (2.7%)
Within normal range	36/37 (97.3%)
**Specific infectious diseases**	**Number / Total (%)**
Influenza	4/37 (10.8%)
HHV-6	6/36 (16.2%)
HHV-7	26/36 (72.2%)
**Medication for IM symptoms**	**Number / Total (%)**
Antibiotics	22/37 (59.46%)
Steroids	3/37 (8.1%)
**Medical history within 6 months before IM onset**	**Number / Total (%)**
Fever	17/37 (46.0%)
Travelling to Europe/the Middle East	22/37 (59.5%)
Travelling Far East	1/37 (2.7%)
Antibiotics	4/37 (10.8%)
Steroids (oral, intravenous)	2/37 (5.4%)
Analgetics/antiphlogistics	14/37 (37.8%)
**Others**	**Number / Total (%)**
Allergies	8/37 (21.6%)

Asthma	1/37 (2.7%)
**Clinical diagnostic tools**	**Number / Total (%)**
Mc Isaac Score = 0	0 /37 (0.0%)
Mc Isaac Score = 1	3/37 (8.1%)
Mc Isaac Score = 2	6/37 (16.2%)
Mc Isaac Score = 3	11/37 (29.7%)
Mc Isaac Score $\geq$ 4	17/37 (46.0%)
**Signs of primary immunodeficiency**	**Number / Total** (%)
Known primary immunodeficiency	0/37 (0.0%)
ELVIS - at least one criterion[3]	22/37 (59.5%)
ELVIS - one criterion	19/37 (51.4%)
ELVIS - two criteria	3/37 (8.1%)
ELVIS - unusual pathogen	3/37 (8.1%)
ELVIS - multiple localization	1/37 (2.7%)
ELVIS - relapsing course of infection	9/37 (24.3%)
ELVIS - unusual intensity	7/37 (18.9%)
ELVIS - increased sum of minor infections	5/37 (13.5%)
GARFIELD – at least one criterion[3]	6/37 (16.2%)
GARFIELD - one criterion	6/37 (16.2%)
GARFIELD - granuloma	0/37 (0.0%)
GARFIELD - autoimmunity	1/37 (2.7%)
GARFIELD - recurring fever	3/37 (8.1%)
GARFIELD - eczema, including neurodermatitis	1/37 (2.7%)
GARFIELD - lymphoproliferation	1/37 (2.7%)
GARFIELD - chronic inflammatory bowel disease	0/37 (0.0%
**Medical history of the family**	**Number / Total (%)**
Consanguineous parents	1/37 (2.7%)
At least one sibling in the same household	31/37 (83.8%)
1 sibling	23/37 (62.2%)
2 siblings	6/37 (16.2%)
≥ 3 siblings	2/37 (5.4%)
Cancer	23/37 (62.2%)
Neurodermatitis	7/37 (18.9%)
Allergies	28/37 (75.7%)

Asthma	1/37 (2.7%)
Infectious mononucleosis	10/37 (27.0%)
Classical autoimmune diseases	9/37 (24.3%)
[1] Vaccination status according to the recommendations of the Standing Committee on vaccination (STIKO) at the Robert Koch Institute (RKI), a department of the German Federal Health Agency [50], accessed at the beginning of patient recruitment on 11 February 2006.<br>[2]Normal range of vaccination titers according to the information of the Standing Committee on vaccination (STIKO) at the Robert Koch Institute (RKI), a department of the German Federal Health Agency [51], accessed on 6 February 2024.<br>[3][52]	

SUPPLEMENTARY TABLE S3. Presence of IM Features at Study Visits.

**IM features**	**Number / Total (%)**		
**IM clinical features**	**Visit 1**	**Visit 2**	**Visit 3**
Fever	7/36 (19.4%)	1/36 (2.8%)	0/36 (0.0%)
Fatigue	32/36 (88.9%)	23/36 (63.9%)	7/36 (19.4%)
Lymphadenopathy	28/33 (84.9%)	11/33 (33.3%)	2/33 (6.1%)
Tonsillopharyngitis	28/34 (82.4%)	3/34 (8.8%)	1/34 (2.9%)
Facial edema	14/37 (37.8%)	0/37 (0.0%)	1/37 (2.7%)
Exanthema	3/31 (9.7%)	1/31 (3.2%)	0/31 (0.0%)
Other airway symptoms	19/28 (67.9%)	4/28 (14.3%)	2/28 (7.1%)
Other gastrointestinal symptoms	14/34 (41.2%)	2/34 (5.9%)	1/34 (2.9%)
Urogenital symptoms	1/36 (2.8%)	1/36 (2.8%)	0/36 (0.0%)
Neurological symptoms	13/36 (36.1%)	4/36 (11.1%)	4/36 (11.1%)
Musculoskeletal symptoms	6/36 (16.7%)	1/36 (2.8%)	2/36 (5.6%)
Hospitalization	13/37 (35.1%)	0/37 (0.0%)	1/37 (2.7%)
**IM laboratory features**	**Visit 1**	**Visit 2**	**Visit 3**
Lymphocytosis	2/37 (5.4%)	0/37 (0.0%)	0/37 (0.0%)
Neutropenia	7/37 (18.9%)	5/37 (13.5%)	0/37 (0.0%)
Anemia	1/36 (2.8%)	0/36 (0.0%)	0/36 (0.0%)
Thrombocytopenia	5/37 (13.5%)	1/37 (2.7%)	1/37 (2.7%)
Hyperferritinemia	15/36 (41.7%)	1/36 (2.8%)	0/36 (0.0%)
Hepatitis or cholangitis	21/35 (60.0%)	6/35 (17.1%)	0/35 (0.0%)
Elevation of C-reactive protein	13/37 (35.1%)	3/37 (8.1%)	0/37 (0.0%)
**IM-complexity clinical**	**Visit 1**	**Visit 2**	**Visit 3**
0 IM clinical feature	1/37 (2.7%)	6/37 (16.2%)	26/37 (70.3%)
1 IM clinical feature	0/37 (0.0%)	9/37 (24.3%)	7/37 (18.9%)
2 IM clinical features	3/37 (8.1%)	12/37 (32.4%)	1/37 (2.7%)
≥ 3 IM clinical features	33/37 (89.2%)	10/37 (27.0%)	3/37 (8.1%)
**IM-complexity laboratory**	**Visit 1**	**Visit 2**	**Visit 3**
0 IM laboratory feature	5/37 (13.5%)	24/37 (64.9%)	36/37 (97.3%)

1 IM laboratory feature	12/37 (32.4%)	10/37 (27.0%)	1/37 (2.7%)
2 IM laboratory features	11/37 (29.7%)	3/37 (8.1%)	0/37 (0.0%)
≥ 3 IM laboratory features	9/37 (24.3%)	0/37 (0.0%)	0/37 (0.0%)
**IM-complexity total**	**Visit 1**	**Visit 2**	**Visit 3**
0 IM feature	1/37 (2.7%)	5/37 (13.5%)	26/37 (70.3%)
1 IM feature	0/37 (0.0%)	5/37 (13.5%)	7/37 (18.9%)
2 IM features	1/37 (2.7%)	13/37 (35.1%)	1/37 (2.7%)
≥ 3 IM features	35/37 (94.6%)	14/37 (37.8%)	3/37 (8.1%)
**IM-severity clinical**[1]	**Visit 1**	**Visit 2**	**Visit 3**
S0	1/37 (2.7%)	6/37 (16.2%)	26/37 (70.3%)
S1	12/37 (32.4%)	27/37 (73.0%)	8/37 (21.6%)
S2	23/37 (62.2%)	4/37 (10.8%)	2/37 (5.4%)
S3	1/37 (2.7%)	0/37 (0.0%)	1/37 (2.7%)
**IM-severity laboratory**[1]	**Visit 1**	**Visit 2**	**Visit 3**
S0	5/37 (13.5%)	24/37 (64.9%)	36/37 (97.3%)
S1	22/37 (59.5%)	11/37 (29.7%)	1/37 (2.7%)
S2	10/37 (27.0%)	2/37 (5.4%)	0/37 (0.0%)
**IM-severity total**[1]	**Visit 1**	**Visit 2**	**Visit 3**
S0	1/37 (2.7%)	5/37 (13.5%)	26/37 (70.3%)
S1	9/37 (24.3%)	26/37 (70.3%)	8/37 (21.6%)
S2	26/37 (70.3%)	6/37 (16.2%)	2/37(5.4%)
S3	1/37 (2.7%)	0/37 (0.0%)	1/37 (2.7%)
**IM-protraction**	**Visit 1**	**Visit 2**	**Visit 3**
Symptoms at visit 3[2]	n/a	n/a	9/37 (24.3%)

[1]The severity levels are described elsewhere [28]
[2]Except S1 for Lymphadenopathy and/or splenomegaly
n/a: not applicable

SUPPLEMENTARY TABLE S4. Other laboratory features at study visits.

Other laboratory features	Mean (SD) [N]		
	Visit 1	Visit 2	Visit 3
Leucocytes (G/l)	11.2 (5.3) [N = 37]	6.9 (2.5) [N = 37]	7.3 (3.3) [N = 37]
Hemoglobin (g/dl)	12.9 (1.6) [N = 37]	12.7 (1.2) [N = 37]	13.4 (1.3) [N = 37]
Thrombocytes (G/l)	247.5 (97.6) [N = 37]	277.6 (66.9) [N = 37]	287.2 (81.5) [N=37]
Neutrophils (%)	25.4 (10.7) [N = 37]	41.6 (9.3) [N = 37]	51.0 (10.1) [N=37]
Lymphocytes (%)	37.6 (12.4) [N = 37]	43.1 (8.5) [N = 37]	36.6 (9.6) [N=37]
Monocytes (%)	5.8 (3.8) [N = 37]	8.9 (2.3) [N = 37]	7.8 (1.8) [N=37]
Creatinine (mg/dl)	0.6 (0.2) [N = 37]	0.6 (0.2) [N = 37]	0.6 (0.2) [N=37]
CrP (mg/dl)	1.2 (1.6) [N = 37]	0.4 (1.6) [N = 37]	0.1 (0.1) [N=37]
GPT (ALAT) (U/l)	149.8 (146.6) [N = 37]	46.1 (46.7) [N = 37]	19.4 (9.6) [N = 37]
GOT (ASAT) (U/l)	104.3 (89.2) [N = 36]	37.7 (17.1) [N =36]	29.4 (11.4) [N=36]
Ferritin (ng/ml)	214.5 (230.4) [N = 36]	56.5 (40.5) [N = 36]	36.2 (20.2) [N = 36]

SUPPLEMENTARY TABLE S5. Virological IM features at study visits.

Virological IM features	Number / Total (%)		
	Visit 1	Visit 2	Visit 3
PCR: EBV DNA in cell fraction	34/34 (100.0%)	26/34 (76.5%)	25/34 (73.5%)
PCR: EBV DNA in plasma	30/36 (83.3%)	4/36 (11.1%)	4/36 (11.1%)
PCR: EBV DNA in oral wash	22/22 (100.0%)	17/22 (77.3%)	10/22 (45.5%)
CMIA: EBV VCA lgM	31/32 (96.9%)	31/32 (96.9%)	18/32 (56.3%)
CMIA: EBV VCA lgG	30/32 (93.8%)	30/32 (93.8%)	32/32 (100.0%)
CMIA: EBNA1 IgG	2/32 (6.3%)	2/32 (6.3%)	27/32 (84.4%)
Immunoblot: EBV EAp54 IgG	28/32 (87.5%)	28/32 (87.5%)	24/32 (75.0%)
Immunoblot: EBV EAp138 IgG	17/32 (53.1%)	16/32 (50.0%)	24/32 (75.0%)
Immunoblot: EBV BZLF1 IgG	27/32 (84.4%)	29/32 (90.6%)	29/32 (90.6%)
Immunoblot: EBV VCAp23 IgG	15/32 (46.9%)	17/32 (53.1%)	32/32 (100.0%)
Immunoblot: EBV VCAp18 IgG	6/32 (18.8%)	8/32 (25.0%)	32/32 (100.0%)
Immunoblot: EBNA1 IgG	1/32 (3.1%)	1/32 (3.1%)	25/32 (78.1%)
